# Costal Osteoid Osteoma: A Case Report and Review of the Literature

**DOI:** 10.7759/cureus.85589

**Published:** 2025-06-09

**Authors:** Afnane Ismaili, Anass Chbhi-Kaddouri, Ilyass Chergaoui, Anass Kherrab, Mirieme Ghazi, Redouane Niamane

**Affiliations:** 1 Rheumatology Department, CHU Mohammed VI, Cadi Ayyad University, Marrakesh, MAR; 2 Rheumatology Department, Avicenne Military Hospital, Cadi Ayyad University, Marrakesh, MAR

**Keywords:** costal resection, neuropathic pain, osteoid osteoma, rib, thoracotomy

## Abstract

Costal osteoid osteoma is a rare benign tumor that can be diagnostically challenging due to its atypical location and nonspecific symptoms. We report the case of a 49-year-old male presenting with chronic right-sided chest wall pain for three years. Initial radiographs were normal, but CT imaging and bone scintigraphy later revealed a focal lesion on the ninth right rib. Surgical resection was performed, confirming the diagnosis histologically. The patient subsequently developed persistent moderate intercostal neuropathic pain. This report illustrates the diagnostic pitfalls of rib-localized osteoid osteoma, particularly when initial imaging is inconclusive, and emphasizes the importance of considering this rare entity in patients with unexplained chronic thoracic pain.

## Introduction

Osteoid osteoma is a benign osteoblastic tumor accounting for approximately 3% of all primary bone tumors [1]. It mainly affects adolescents and young adults, with a male predominance [1-3]. Clinically, it presents as nocturnal pain that typically responds well to nonsteroidal anti-inflammatory drugs (NSAIDs) [1,4]. The tumor most often involves the diaphysis or metaphysis of long bones, particularly the femur and tibia [1,4,5]. Rib involvement is rare, representing less than 1% of all rib tumors [5,6]. When present, it poses a diagnostic challenge and often results in delayed diagnosis due to nonspecific symptoms and atypical imaging findings, such as an inconspicuous nidus or minimal surrounding sclerosis, especially when the lesion is located near neurovascular structures [7].

Although minimally invasive approaches such as CT-guided radiofrequency ablation have emerged as effective alternatives [3], surgical excision remains the mainstay of treatment, particularly when the lesion is close to critical structures or when histopathological confirmation is required. In our case, the lesion's location on the posterior arch of the rib and proximity to neurovascular structures made surgical resection the safer and more definitive option. However, this approach carries a risk of intercostal nerve injury, potentially leading to chronic postoperative neuropathic pain [8]. We present a rare case of costal osteoid osteoma managed by surgical resection, complicated by persistent intercostal neuralgia.

## Case presentation

A 49-year-old male nursing assistant, with a history of Helicobacter pylori gastritis (treated in 1999) and renal lithiasis (treated by extracorporeal lithotripsy in 2013), presented in August 2022 with right-sided chest wall pain evolving over three years. The pain had begun insidiously, without any preceding trauma, and progressively intensified to become almost constant. It had inflammatory features, with nocturnal exacerbations and partial relief with NSAIDs. The pain was described as deep, burning, and localized, sometimes radiating anteriorly, and was aggravated by coughing, deep inspiration, cold exposure, and certain movements of the right upper limb, particularly elevation.

**Figure 1:**
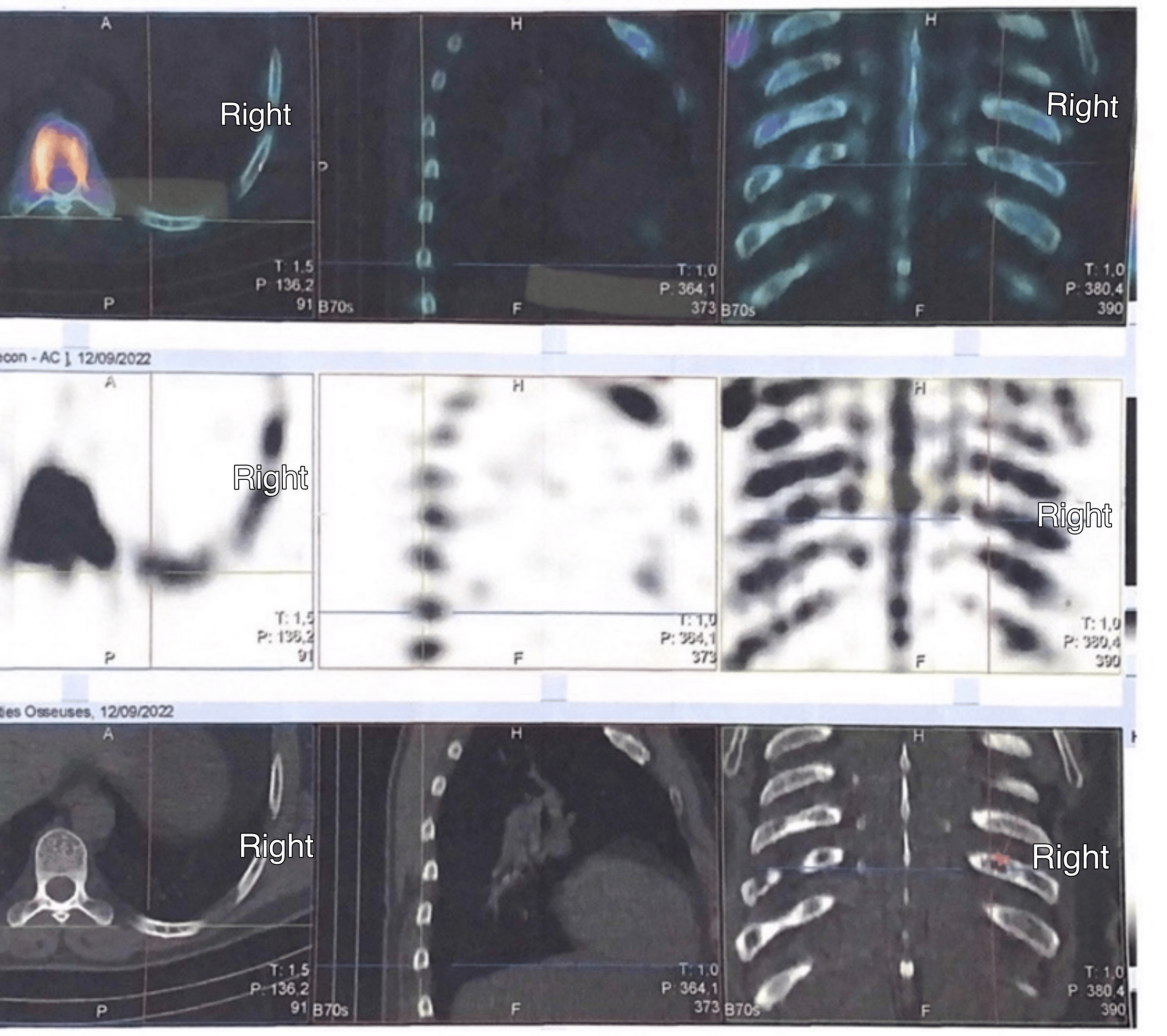
CT findings (axial, coronal, and sagittal views) The images show a sclerotic lesion of the posterior arch of the ninth right rib without cortical breach, suggestive of osteoid osteoma CT: computed tomography

**Figure 2:**
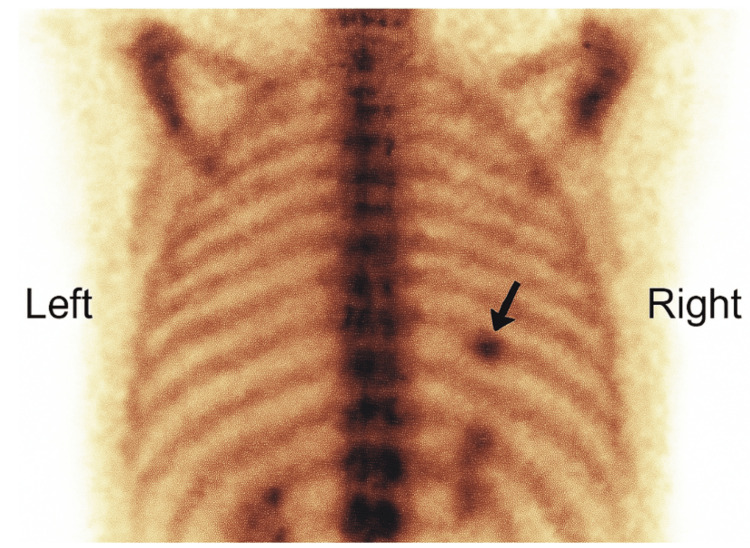
Bone scintigraphy showing focal hyperfixation at the posterior arch of the ninth right rib

The patient presented to the emergency department on three separate occasions during the first two years. No clear diagnosis was established, and treatment was limited to analgesics. NSAIDs were initially effective, but with time, only aspirin provided sustained pain relief. Clinical examination revealed localized tenderness on deep palpation over the 9th-10th right ribs at the posterior axillary line. Right arm elevation beyond 90 degrees reproduced the pain, suggesting irritation by mechanical mobilization of the lower scapulothoracic region. Standard chest X-rays and costal series were unremarkable. CT scan showed a sclerotic lesion on the posterior arch of the 9th right rib without cortical breach, suggestive of osteoid osteoma (Figure 1). A two-phase bone scintigraphy confirmed hyperfixation at the same site (Figure 2).

Surgical referral led to a partial resection of the ninth rib via right posterolateral thoracotomy. This approach was chosen due to the lesion’s location near the costovertebral angle, which rendered percutaneous ablation technically difficult. Histopathological analysis confirmed osteoid osteoma.

On postoperative day one, the patient developed unilateral intercostal neuropathic pain along the incision site, described as a burning sensation with hyperesthesia, consistent with intercostal nerve injury. He required 10 days of hospitalization with strong opioid therapy. After 12 months, moderate chronic neuropathic pain persists, worsened by coughing, cold, and certain movements. He is not undergoing any treatment currently.

## Discussion

First described by Henry Louis Jaffe in 1935, osteoid osteomas typically affect the diaphysis of long bones, particularly the lower limbs [9]. Rib involvement is exceptionally rare and often mimics malignant tumors such as metastases or myeloma [6,7,10]. Rib osteoid osteomas represent only 0.23% to 2% of all cases [11]. Only a few cases of costal osteoid osteoma have been reported in the literature. In the case reported by Deng et al. [5], diagnosis was delayed due to nonspecific symptoms and unremarkable initial imaging. Similarly, Hughes et al. [11] highlighted the rarity of benign rib tumors and the frequent diagnostic confusion with malignancies. Our report reinforces these findings, emphasizing the importance of considering osteoid osteoma in patients with chronic localized thoracic pain and negative standard radiographs.

Diagnosis is difficult due to the rarity and lack of typical radiographic features in 25% of cases [9]. Standard X-rays may be inconclusive, especially when extensive peripheral osteosclerosis masks the nidus [12]. In our case, the lesion was invisible on initial radiographs, delaying diagnosis. CT scan remains the key imaging modality, enabling precise localization, especially in complex anatomical regions. Scintigraphy is highly sensitive, showing intense uptake in the hypervascularized nidus and surrounding reactive sclerosis [12]. Surgical excision is the gold standard for treatment, enabling both histological confirmation and rapid symptom relief [1,5,13]. Minimally invasive alternatives such as radiofrequency ablation, laser ablation, and cryoablation are increasingly used depending on lesion size, location, equipment availability, and practitioner preference [12]. In our case, conventional thoracotomy was necessary. However, such approaches are highly invasive and are known to cause chronic neuropathic pain. The incidence of post-thoracotomy chronic pain varies from 30% to 50% [8,14]. Katz et al. reported that acute pain intensity after thoracic surgery is a predictor of chronic pain [15]. Neuropathic pain affects 22% of patients at two months and 14% at 12 months [14]. Optimal postoperative analgesia, including strong opioids, is crucial. In Guastella et al.’s series, intravenous morphine consumption in the first 72 hours post-op reached up to 70 mg/day [13]. Chronic neuropathic pain is managed with anticonvulsants such as gabapentin or pregabalin, and certain tricyclic antidepressants [16].

This report highlights how the rarity of costal involvement and the nonspecific imaging findings can lead to diagnostic delays, particularly when standard radiographs are inconclusive. Increasing awareness of such atypical presentations may help clinicians refine their differential diagnoses and reduce time to diagnosis in cases of persistent, unexplained thoracic pain.

## Conclusions

Costal osteoid osteoma is an exceedingly rare entity, often leading to delayed diagnosis due to its atypical clinical and radiological presentation. Although minimally invasive techniques have gained popularity in recent years, surgical resection remains the gold standard in anatomically complex cases. However, clinicians must be aware of potential postoperative complications, particularly thoracotomy-related intercostal neuropathic pain, which can significantly impact quality of life and requires appropriate long-term management.
